# Conceptualizing Medical Resident’s Journey through Wonderland of Clinical Practice: From transitional shock to role adaptation to independent practice

**DOI:** 10.12669/pjms.37.4.3853

**Published:** 2021

**Authors:** Marium Sohail, Rahila Yasmeen

**Affiliations:** 1 Dr. Marium Sohail, MBBS, MHPE. Assistant Professor / Director, Department of Medical Education, Poonch Medical College, Rawalakot, AJ&K, Pakistan; 2 Dr. Rahila Yasmeen, BDS, DCPS- HPE, MHPE, PhD-HPE (Scholar). Professor/ Director, Department of Medical Education, DEAN RARE/ ORIC, Riphah International University, Islamabad, Pakistan

**Keywords:** Education, Medical, Residency, Learning, Transitional journey

## Abstract

**Objectives:**

To explore experiences of medical residents about stages and nature of transitional journey from induction into residency program to achievement of consultant title.

**Methods:**

Qualitative phenomenological study was conducted at Sir Gangaram hospital and Fatima Memorial Hospital, Lahore for six months from Feb 2019-July 2019 after IRB/ERC approval from Islamic International Medical College (Ref# Riphah/IIMC/ERC/19/0320). Using purposive criterion sampling, 16 semi-structured interviews in four departments, four strata of junior residents, senior residents, newly-qualified consultants, and supervising consultants with four participants each were conducted till theoretical saturation of data was achieved. After informed consent, audio recorded data was transcribed verbatim & analyzed through Atlas.ti 7 using Interpretive Phenomenological Analysis. After Bracketing and horizontalization, data was coded line by line. Codes (324) were merged to categories (19) for structural and textural description. Composite description of phenomenon was done by abstraction of themes (eight for stages and two for nature of journey).

**Results:**

Eight themes for stages as merriment, anguish, melancholy, acceptance and efforts, intensive learning, burnishing to shine, identity formation and intra-stage oscillations were identified. Two themes for journey’s nature were exponential learning & oscillating journey.

**Conclusion:**

The transitional journey is a multi-stage complex, oscillating journey. An oscillating electron model is presented upon the findings of this study to explain resident transition

## INTRODUCTION

Transition, in context to medical education refers to movement between different phases of medical training i.e. undergraduate, postgraduate and continuing medical education. Transitional shock is defined as “a crisis experienced by health professional and is marked by a temporary decrease in their ability to properly use biomedical knowledge in clinical reasoning and practice”.^1^ Role adaptation refers to “acclimatization to transition or role in which an individual moves from being totally preoccupied with transition to integrating transition into his life by a changing one’s behavior and attitude”.^2^

Transition across different facets of educational, psychological and socio-cultural variations have a huge impact on identity formation and development of sense about one’s self.^3^ Transition is a personalized endeavor but is challenging and stressful as one enters into a new learning environment.^4^,^5^ Worldwide studies indicate an increasing trend in negative effects like anxiety, depression and burnout in doctors.^6^ By the time physicians reach residency, rates of depression are four times the national average.^7^ One of every three budding physicians experiences an episode of major depression during their post graduate training.^7^ A recent rise in rate of suicide among Pakistani doctors with a greater propensity during residency period has been observed.^8^

Though the changes in responsibilities occur at every level of transition, yet the transition to “independent consultant practice” poses to be the most stressful because of the implicit assumption of the educators and clinicians that postgraduate medical students are “expert students” as they have successfully gained undergraduate degree, acquired residency position & environment hasn’t changed so their knowledge is continued and not re-situated.^9^,^10^ This assumption does not hold true as Postgraduate students are a heterogeneous and not a homogenous community & all variables cannot be just negated and a general assumption be made for post graduate trainees.^9^

Stephens described objective career as externally defined reality of the career that comprise of person’s work history while subjective career is typified in the attitudes, orientations, and perceptions about the career that are held by an individual.^11^ So, it’s very important that subjective and objective career complement each other in order to make the transition between the professional stages easier, manageable and successful for health professional.

So, transition is conceptualized as a holistic and dynamic amalgamation of educational, psychological and socio-cultural variations.^12^ Conceptualizing progress of journey through insight into experience of residents in transitional period would be valuable for medical educators to identify the stages of journey and hence help in designing support interventions.

### Research question

What are Medical resident’s lived experiences about the stages of journey from transitional shock to role adaptation in clinical practice?

## METHODS

**Fig.1:**
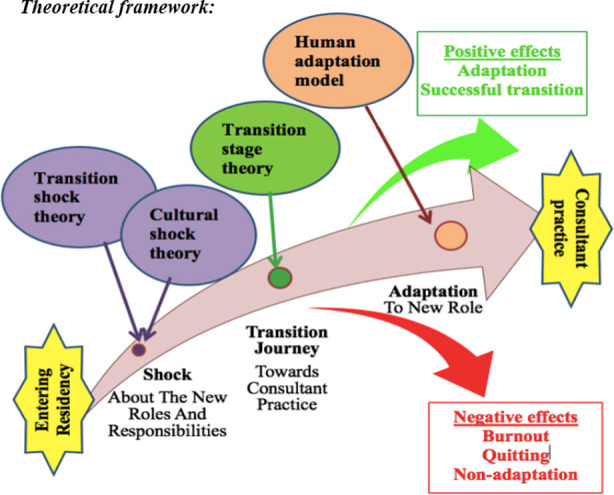
Theoretical framework for Transition phenomenon.^2^,^13^,^14^

A qualitative phenomenological study was conducted at Sir Gangaram Hospital and Fatima Memorial Hospital, Lahore for six months from February 2019 - July 2019. Interview guide was constructed using theoretical framework (Fig.1), validated from experts and piloted prior to use. After Ethical approval, from IRB/ERC of Islamic International Medical College (Ref# Riphah/IIMC/ERC/19/0320). using purposive criterion sampling, 16 semi-structured interviews were conducted till theoretical data saturation was achieved. Four different clinical departments (medicine, surgery, gynecology and pediatrics), in four strata of junior residents, senior residents, newly-qualified and supervising consultants were involved in the study with four participants each. The interview was same for all strata, but prompts use for exploring different phases of transitional journey were different. After informed consent, audio recorded data was transcribed verbatim using anonymous names. Researcher also took notes of nonverbal cues during interviews in addition to audio recording. Excerpts in English were written as such while those in Urdu were translated by an expert in English language. Concurrent data analysis was done in order to modify data collection process and include the emerging themes in subsequent interviews. Data was analyzed through Atlas.ti 7 by Interpretive Phenomenological Analysis using codes from both analytical framework and in-vivo coding. After Bracketing and horizontalization, data was coded line by line. Codes (324) were merged to categories (19) for structural and textural description. Finally, the composite description of phenomenon was done by abstraction into eight themes outlining the stages & two outlining nature of transitional journey.

**Table-I:** Stages of transitional journey with representative quotes

Sr.No	Theme/ (Frequency)	Category / Subtheme	Code/ (frequency)	Representative Quote
**Stages of Journey**				
1	Delectation & Merriment (31)	Dream come true	Happiness (12)	*“I screamed, and I jumped and hugged my family and it was a very different day… It’s like you’ve achieved the greatest thing….something like that.” **C2***
		Achievement	Accomplishment (19)	
2	Anguish (74)	Uncertainty	Confusion and Distress (25)	*“… it was simply too much to take, I didn’t know what to do, how to do and when to do? I thought I wasn’t capable of doing all this, it’s not my type…”**A2***
		Lacking clarity	Self-doubt (18)	
		Reality check	Discrepancy in reality vs. expectations (21)	*“…life is tough and I have to work damn hard. Those bed of roses vanished so suddenly, its different to What I imagined …”**C3***
		Fear of medical malpractice	Afraid of negligence in patient care (10)	*“I am accountable for patient. And that’s not a small thing, that’s a life in my hand…” **A1***
3	Melancholy (23)	Shock and depression	Newness is scary (8)	*“I was afraid, I felt depressed coz everything felt new, no friends, new place and me being naïve and depressed”**C2*** *“…, I panicked and started crying, I shouted at nurse to save him; …”**D3***
		Emotional instability	Panic Attacks (11)	
		Turbulence	Crying (5)	
4	Acceptance & Dedicated Efforts (104)	Self-realization	Acceptance of short comings (35)	*“…I am lacking in my knowledge and my clinical skills, and I need to improve if I wish to survive”**A2***
		Creating support system around	Create comfort zone by seeking help (18)	*“When I started to develop my rapport and good relationship in the unit…, I started to feel relaxed.” **B3*** *.“… I regained rationality, from that day onward, I started living my dream…”**B2***
		Positive attitude	Learning to survive (29)	
		Recognizing and seizing learning avenues	Voluntary efforts for learning (22)	*“… I was always asking questions & searching for answers” **A2***
5	Intensive learning (67)	Pacing up learning	Eagerness to use opportunities to fullest (32)	*“It’s a dark tunnel with shining light at the end but you have to look for the torches yourself to go to that end, I used to learn from where ever I can, I used to look around for someone willing to teach me, I even went to other hospitals to study…**C3***
		Intensive dedicated efforts for learning	Voluntary involvement in opportunities (35)	
6	Burnish to shine (38)	Recognizing facets for improvement	Acceptance of limitations (9)	*“One should always know his limitations, no one knows it all, everyone has finite knowledge, if you don’t know, seek help, get someone on board, learn for next time”**D3***
		Polishing to shine	Working on weaknesses (14)	
		Self-efficacy and self-regulation	Periodic self-assessment to drive learning (15)	*“…You should know where to fine tune and where to initiate crises management, YOU can only win, if u speak truth to yourself” **D4***
7	Identity formation (41)	Gaining insight	Broadening of professional mission and vision (15)	*“it’s about equipping oneself with specialized knowledge, skills and combat strategies for emergent patient need and personal professional growth to make your own specialized worth in healthcare setup and society” **D3***
		Establishing distinct individualization	Becoming Real doctors (26)	
8	Intra stage oscillation at exam check point (48)	Crises due to stress of IMM	Shift of focus from patient to exam due to stress of IMM (29)	*“I was so depressed before my IMM exam; I used to study all night and was sleepy during my duty hours. My concentration shifted from patients to passing exam, I was stressed and I still feel guilty about it” **C3***
		Burn Out before Part 2 exam	Exhausted and depressed near Part 2 exam (19)	

## RESULTS

Sixteen members from four departments were divided into four groups on the basis of year of training. Of all the respondents 44% (7) were males while 56% (9) females. The average age of trainees was 30.9 years while that of supervisors was 55.3 years.

The residents identified eight stages of transition journey, Delectation and merriment followed by anguish, melancholy which then translates to acceptance and dedicated efforts, intensive learning, burnishing to shine and finally identity formation. However, residents experience intra stage oscillations at various points during the journey especially at exam check points. The residents described that there are two dimensions of journey as a doctor. One is the learning dimension which always increases exponentially while the other is the emotional dimension which is turbulent and oscillating. The nature of journey overall is determined more by the emotional dimension as it drastically effects on the learning and application of learning dimension.

Gender wise comparison between perspectives shows stages of melancholy, acceptance of reality, establishing self-efficacy and developing positive attitude towards learning were more strongly represented in female population while stages of lacking clarity anguish, creating support system and identity formation was reported more strongly in male population. The type of journey perceived as linear or exponential for learning and oscillating overall did not vary across gender.

Comparison of perspectives with respect to years of experience shows no significant difference in the stages of journey perception across different strata of years of experience. Those in initial years of experience had no idea about the later stages, they only gave the life experiences of the stage they are in. However, the resident’s with more than three years of experience gave rich information about both early and late stages.

## DISCUSSION

**Fig.2:**
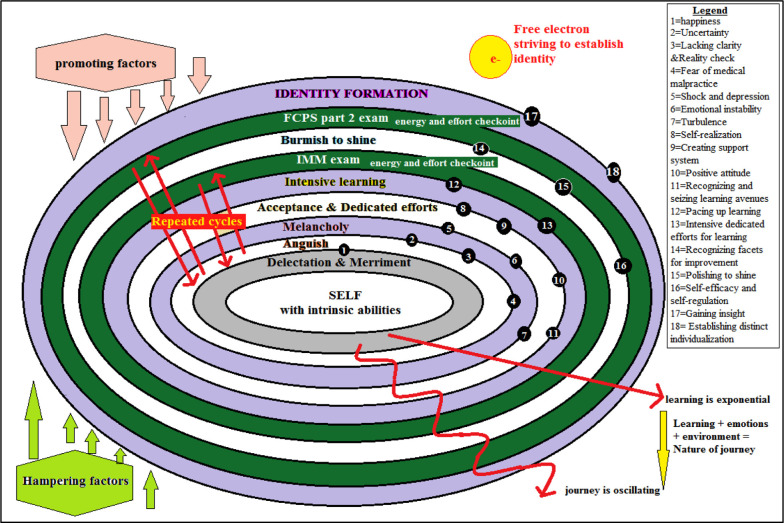
Sohail’s oscillating electron model for transition.

Eight stages for journey & two for nature of journey are represented as Sohail’s Oscillating electron model (Fig.2). This study has various similarities and differences across different spectrum of studies. Some stages are overlapping to those reported in literature while some sub stages are distinctive. Draper J reported that transitional path is individualized yet variable & similar milestones across transitional journey can be identified despite of contextual differences.^15^ Wegner and Snyder Communities of practice theory states that people with similar domain of interest, works together to share ideas and practice which ultimately leads to improved practice in the domain, as they learn how to do better what they are doing. The life cycle of CoP has potential, coalescing, maturing, active, dispersed/repository stages.^16^ The pattern of emotions experienced by doctors in this study align clearly with the life events transition reported by Fisher, Porteous, Wall and Duschers adding validity to the findings and reinforcing the results of my study.^13^.^14^.^17^,^18^ Hopson’s reported stages of excitement & honeymoon, uncertainty, depression and crises, quitting or letting go, partial recovery, exploration, nesting and ultimately transformation in a linear graphical form with positive and negative deflections.^19^ Fisher reported Anxiety, happiness, threat, guilt, depression, gradual acceptance and moving forwards, while Porteous identified uncertainty, learning to survive, looking out for help and moving forwards as important stages.^17^,^20^ Getting ready to move, moving in, organizing to move out and moving out were identified by Wall as four distinct features of transitional journey.^21^ Draper also highlighted similar stages in his study on nurses.^15^ Hardy and nightingale reported phases of elation, denial, doubt, crises and recovery for radiographic technicians.^22^ As per **Duchscher** the nurses move from Doing to Being to Knowing stage.^13^.^14^ After transition shock the nurses recover, learn and re-learn to reveal true potential within self. The final stage is to know and accept the real self. This linear trajectory of experiential learning corresponds to the stages of acceptance, intensive learning, polishing to shine and identity formation in my study. The sub-stages of fear of medical malpractice, self-reflection for realization and gaining insight are distinctive in my study.

**Table-II:** Nature of Transition Journey with representative quotes

Findings of Nature of Transition Journey from the Data				
S/N	Theme	Category / Subtheme	Code	Representative Quote
1	Learning is exponential (23)	Experiences drives learning	Learning from experiences (8)	*“Whenever you are thrown out to experience something, you learn… learning is invisible”**D2*** *“Someday you have an adrenaline rush and learn more and then there are days when you nearly learn nothing because of your laziness. But even laziness teaches you what not to do… laughter.” **B3***
		Wanted and unwanted learning	Good or bad, you always learn (6)	
		Formal and informal learning	Learning occurs everywhere (5)	
		Pace of learning changes	You learn: more or less but never NO (4)	
2	Journey is oscillating (34)	Back and forth process	Bouncing back and fro (9)	*“I think it’s a way forward journey towards your goals with turbulences here and there”**B4*** *“The process is linear for learning and spring like motion for emotional journey. the more you put pressure the more you bounce back…. (laughter)” **A2***
			Ups and downs (6) Way forward with turbulence (5)	
			Bouncing spring (10)	
		Pendulum like journey	Pendulous (4)	*“it’s like a pendulum like journey, here to there, there to here, till you reach 12 to strike and stay”* ***D3***

Draper & Maria stated that transition is an organic journey with varied experiences, variable expressions and an individual endeavor with indefinite boundaries.^15^,^23^ Colbert-getz states that experiences vary which causes fluctuating states of emotional, physical and social instability.^24^ All these studies have similar perspectives explained as in this study as an oscillating journey with a net forward direct for learning. The oscillation during the transitional journey is due to intrinsic and extrinsic factors playing in the picture. In contrast to it, Duchscher identifies a linear trajectory of experiences for nurses in his study.^14^ He explained the journey in equivalence to time to regain their rationality.^13^ This study gives a contrasting view and may be attributed to the difference in the context and working of doctors and nurses.^25^

The oscillating electron model (Fig.2) depicts oscillating movement across the shells which keeps electron moving up and down with the difference in energy i.e. the resident oscillates between stages with the presence of extent of promoting and hampering factors. If hampering factors are more backward oscillations are more. If promoting factors are strong forward oscillations are strong. The learning is exponential and always increases while the journey is oscillating.

It is a qualitative study hence its non-generalizable to wider audience. Such a study has never been conducted for medical or dental doctors hence comparison of results from similar context is unavailable. Due to time constraints lived experiences were explored retrospectively only. Longitudinal exploration can be done for re-verification and triangulation of findings. Educational interventions can be planned using oscillating electron model. The study can be conducted across different cultures, strata and specialties to establish a comparison.

## CONCLUSION

The stages identified for the resident’s transitional journey are merriment, anguish, melancholy, acceptance and efforts, intensive learning, burnishing to shine and identity formation. Interspaced among these stages are two checkpoints for the learning, effort and energy assessment. The residents oscillate between stages and sub-stages; some stages might be skipped for each individual depending upon their context. Learning is exponential while the journey is perceived to be oscillating. An oscillating electron model is presented to explain the resident transition.

### Authors Contribution:

**MS:** Conceived, designed, collected data, did phenomenological analysis, writing, editing of manuscript and is responsible for integrity of the study. **RY:** Supervised, did review and final approval of manuscript.
